# Affective and cognitive components of students’ attitudes towards communication learning - validation of the Communication Skills Attitude Scale in a cohort of polish medical students

**DOI:** 10.1186/s12909-021-02626-7

**Published:** 2021-04-01

**Authors:** Piotr Przymuszała, Magdalena Cerbin-Koczorowska, Patrycja Marciniak-Stępak, Łucja Zielińska-Tomczak, Martyna Piszczek, Jan Jasiński, Ryszard Marciniak

**Affiliations:** 1 Chair and Department of Medical Education, Poznan University of Medical Sciences, 7 Rokietnicka St, 60-806 Poznan, Poland; 2 Department of Pediatric Oncology, Hematology and Transplantology, Poznan University of Medical Sciences, Poznan, Poland; 3 Students’ Scientific Club of Medical Education, Chair and Department of Medical Education, Poznan University of Medical Sciences, Poznan, Poland

**Keywords:** Communication skills attitude scale, CSAS, Medical students, Affective components of attitudes, Cognitive components of attitudes

## Abstract

**Background:**

The Communication Skills Attitude Scale (CSAS) is a recognized tool for assessment of attitudes towards communication learning. In the original version, it consists of 26 items divided on theoretical assumptions into two subscales: Positive and Negative Attitudes Scales. However, the evidence for its structure seems unsatisfactory, and a simple division into positive and negative attitudes may be insufficient to describe attitudes of medical students towards communication learning. Moreover, the existing evidence of the test-retest reliability of the CSAS seems limited. Consequently, this study aimed to provide more evidence on its psychometric properties while validating the CSAS questionnaire in a cohort of Polish medical students.

**Methods:**

The CSAS was translated, adapted into Polish, and validated in a cohort of 389 Polish medical students. Statistical analysis involved, among others, parallel analysis to determine the number of factors, confirmatory factor analysis to compare the proposed model with theory-based ones, and test-retest reliability analysis.

**Results:**

Conducted analysis revealed that in the examined population, the CSAS should rather consist of four than two subscales. Proposed four subscales addressed perceived outcomes of communication learning, positive and negative attitudes towards it (affective components), and factors motivating students to learn communication (a cognitive component of attitudes). Results of test-retest reliability were satisfactory for individual items and subscales.

**Conclusions:**

This study presented a valid and reliable version of the Communication Skills Attitude Scale for Polish medical students and confirmed previous assumptions that CSAS may also be appropriate for assessment of affective and cognitive components of attitudes. Future research should, based on Ajzen’s Theory of Planned Behavior, make attempts to develop a tool assessing not only attitudes but also subjective norms and perceived behavioral control.

**Supplementary Information:**

The online version contains supplementary material available at 10.1186/s12909-021-02626-7.

## Background

Good communication skills of physicians have been linked to increased patients’ satisfaction, reduced numbers of malpractice suits, and better outcomes of the therapeutic process [1–5]. They have become one of the vital skills of contemporary doctors [2–4, 6], and many medical schools and universities have incorporated their teaching into medical curricula [7]. Meanwhile, students’ attitudes towards the topic seem to play an important role in the educational process. Medical students, as adult learners, need to know why they should learn communication skills and be internally motivated to do so [8, 9]. Consequently, communication skills training should involve not only the transfer of theory and skills but also shaping students’ beliefs and attitudes to increase the likelihood of them using acquired skills in their future practice. According to Ajzen’s Theory of Planned Behavior, undertaking given activity (behavior) is a direct result of an intention that is influenced by attitudes towards the behavior, among others [10].

The Communication Skills Attitude Scale (CSAS) is a recognized tool for assessment of attitudes towards communication skills learning. It was developed by Rees et al. [11] in the United Kingdom in 2002 and consists of 26 items scored from 1 (strongly disagree) to 5 (strongly agree). Based on theoretical assumptions, items were divided into two 13-item subscales - Positive Attitudes Scale (PAS) and Negative Attitudes Scale (NAS). The CSAS has already been a subject of several validation studies in different countries, but their results are inconsistent. While researchers from Turkey, Spain, Germany and Malaysia replicated the two-factor solution [12–15], those from Norway, Iran and Korea presented three-factorial [16], four-factorial [17] and five-factorial [18] solutions, respectively. Moreover, even in case of studies confirming the two-factor model, some items landed on opposite subscales than in the original version. A detailed summary of the results of the CSAS validation studies conducted on medical students is presented in Table 1. Authors of the papers mentioned above provided different explanations of potential reasons that may have driven the observed differences. The majority of them pointed to the existence of cultural differences, but they did not elaborate on what they may be. Only Ahn et al. [18] (authors from Korea) gave some examples, for instance developmental stage of communication teaching and learning, Korean students’ low motivation and skepticism of learning communication skills in medical schools, or teaching methods used. Also language differences are often mentioned along with the translation procedure, which even correctly conducted, can change the way respondents understand items. Even slightest changes in wording may affect respondents’ understanding of items, especially when the original scale uses phrases or idioms that cannot be directly translated into the target language. Not without the significance are also the statistical procedures performed. Interestingly, initial factor analysis based on Kaiser criterion (eigenvalues greater than 1) revealed many potential variants of initial solutions in the aforementioned papers: five-factorial [16], six-factorial [11–13, 18], seven-factorial [14] and eight-factorial [15]. However, apart from one research team [15], authors did not determine the number of factors to keep, for instance, using highly recommended Horn’s parallel analysis [19, 20]. Instead, their decisions were mostly based on less accurate methods, like Kaiser criterion, analysis of the scree plot, or theoretical assumptions provided by Rees et al. [11]. Similarly, only a few researchers decided to assess the goodness of fit of their models using the confirmatory factor analysis [14, 17]. Evidence for the test-retest reliability also seems limited as the retesting procedures conducted so far involved small numbers of respondents, namely 20 and 39 [11, 17].

**Table 1:** Comparison of CSAS validation studies on medical students

Authors, Year	Country, language, sample details	Factorial structure (items), Cronbach’s α	Items eliminated	CFA
Rees et al. [11], 2002	The United Kingdom, English, 490 1st and 2nd year medical students	1. PAS (4, 5, 7, 9, 10, 12, 14, 16, 18, 21, 22, 23, 25), α = 0.873 2. NAS (1, 2, 3, 6, 8, 11, 13, 15, 17, 19, 20, 24, 26), α = 0.805	–	NO
Anvik et al. [16], 2007	Norway, Norwegian, 1833 medical students	1. Learning (2, 6, 7, 8, 10, 11, 12, 13, 18, 21, 24, 25, 26) α = 0.861 2. Importance (1, 3, 4, 19, 22) α = 0.532 3. Respecting (5, 9, 14,16), α = 0.775	4 items (15, 17, 20, 23)	NO
Harlak et al. [12], 2008	Turkey, Turkish, 179 1st-5th year medical students	1. PAS (1, 4, 5, 7, 8, 9, 10, 12, 13, 14, 16, 18, 21, 23, 25), α = 0.920 2. NAS (2, 3, 6, 11, 15, 17, 19, 20, 22, 24, 26), α = 0.710	–	NO
Ahn et al. [18], 2009	Korea, Korean, 325 2nd year pre-medical and 3rd year medical students	1. Facilitating interpersonal skills (4, 5, 9, 10, 14, 16), α = 0.752 2. Importance within a medical context (2, 19, 21, 26), α = 0.744 3. Motivation (8, 11, 23, 24), α = 0.680 4. Assessment (3, 22), α = 0.446 5. Overconfidence (13,20), α = 0.496	8 items (1, 6, 7, 12, 15, 17, 18, 25)	NO
Molinuevo and Torrubia [13], 2011	Spain, Catalan, 569 1st year nursing students and 1st and 2nd year medicine students	1. PAS (1, 4, 5, 7, 9, 10, 12, 14, 16, 18, 21, 23, 25) α = 0.830 2. NAS (2, 3, 6, 8, 11, 15, 17, 20, 22, 24), α = 0.640	3 items (13, 19, 26)	NO
Busch et al. [14], 2015	Germany, German, 529 1st, 2nd and 4th year medical students	1. PAS (4, 5, 9, 10, 14, 16, 23), α =?^a^ 2. NAS (2, 6, 7, 11, 12, 15, 17, 19, 21, 24, 25, 26), α =?^a^	7 items (1, 3, 8, 13, 18, 20, 22)	YES
Baharudin et al. [15], 2017	Malaysia, English, 171 1st year medical students	1. PAS (4, 5, 7, 9, 10, 12, 14, 16, 18, 19, 21, 23, 25, 26), α = 0.862 2. NAS (2, 3, 6, 8, 17, 20, 22, 24), α = 0.565	4 items (1, 11, 13, 15)	NO
Yakhforoshha et al. [17], 2018	Iran, Persian, 410 medical students from different levels of training	1. Important in medical context (1, 4, 5, 9, 10, 14, 16, 19, 21, 23, 25), α = 0.86 2. Excuse (2, 6, 8, 15, 18, 26), α = 0.75 3. Learning (7, 12, 13, 17, 24), α = 0.65 4. Overconfidence (3, 20, 22), α = 0.62	1 item (11)	YES

^a^ values are missing due to probable error in the given article

To the best knowledge of authors, there are no validated instruments in Polish assessing attitudes of medical students towards communication learning. Meanwhile, due to the complexity of reasons mentioned above, their factorial structure may be different than in other studies. Apart from language differences and statistical analysis methods, it may also be influenced by the shape of the Polish medical education and healthcare system. From our experience based both on unpublished observations of students during communication classes and conducted surveys, most of our students have positive opinions on communication training. However, its importance does not seem to be emphasized as strongly in the medical curriculum as in some other countries. At most Polish medical schools, communication skills and interpersonal competences are not formally assessed. Moreover, they are not taken into account during the medical school application process. For all those reasons, some students may lack motivation for communication training and not see it as equally important as clinical topics, for instance.

Taking all of the above into consideration, we decided to fill these gaps in the literature and provide more evidence for the CSAS validity and reliability. Additionally, given the lack of validated instruments in Polish assessing attitudes of medical students towards communication learning and the potential differences described above, this study presents the development and validation of the Communication Skills Attitude Scale in a cohort of Polish medical students.

## Methods

### Communication training in polish medical curriculum

In Poland, medical studies are regulated by the Ministry of Science and Higher Education, which describes organizational aspects and intended learning outcomes. Universities are left with autonomy to decide on the details, for instance, the year of study when given topic will be realized or the number of hours dedicated to it, but from our observations the situation seems comparable between different medical schools. At Poznan University of Medical Sciences the number of hours dedicated to communication training is gradually rising, however it still seems low in comparison with other courses. For students described in this study (recruited in the academic year 2019/2020), professionalism and communication training is divided between consecutive study years with 10 h in the first year (‘Introduction to professionalism’), 10 h in the second year (‘Professionalism in the workplace’), 15 h in the third year (‘The art of communication with the patient’), 15 h in the fourth year (‘Patient’s perspective’), 10 h in the fifth year (‘Communication in the therapeutic team’), and 15 h in the sixth year (‘Difficult conversations with patients’). These classes are taught by both physicians and psychologists, and of the 75 h planned in total, 21 h are conducted as lectures, 17 as seminars, and 37 as practical courses with medical simulation and simulated patients. Additionally, in the second year students have a 30-h course on the clinical psychology. It should be also noted that the students described in the study are the first year whose communication competences will be formally assessed as an exam in the third and the sixth year of studies.

### Procedure

For the purpose of this study, a Polish version of the CSAS was developed based on the original English version by Rees et al. [11]. Before the study, the consent of Professor Charlotte Rees was obtained to translate and validate the CSAS on a sample of Polish medical students. The original English version was translated into Polish by two independent translators who then compared their translations and agreed on a final joint version. This joint version was later subjected to a review by a panel of three experts in medical education and communication skills training who compared it with the original version and proposed small corrections to make it better fit Polish conditions. For example, in item 3 Polish equivalent of the untranslatable phrase “fail their medical degree” was changed from one meaning “fail to obtain their medical diploma” to “fail to graduate from medical studies.” This version of the CSAS was subsequently back-translated to English by another two independent translators who had not seen the original version before and did not participate in any of the steps above. Finally, both forward and backward translations were once again reviewed by the experts, until consensus over the final version of Polish CSAS was reached. The final version of the Polish CSAS developed for the purpose of this study is presented in the Additional file 1**.**

Questionnaires additionally contained basic demographic questions (gender, age, and year of study).

As the recommended sample size should be at least 300 respondents [21], all 440 first-year medical students (academic year 2019/2020) of Poznan University of Medical Sciences were invited to participate in the study. Questionnaires (in the form of two identical copies stapled together and consecutively numbered on each page) were distributed before a lecture. Prior to that, the procedure was thoroughly explained to students. They were asked to complete only one copy immediately and return it to the researcher (400 students immediately returned the first copy and 389 of them were completed satisfactorily). Students were also instructed, which was explicitly emphasized twice, to complete the second copy after three to 4 weeks and then return it to the researcher (71 students returned the second copy of the questionnaire after that time). Students were also given the opportunity to ask questions on the procedure in case something was unclear. They were assured that participation in the study was voluntary and that by returning the questionnaire, they agree to take part in it. They were also assured that all data would be obtained and processed anonymously. It should also be emphasized that in order not to put any pressure on students, the lecture was not held by the researcher distributing the questionnaires. Given that the study was not a medical experiment and did not involve patients, the Institutional Bioethical Committee decided that ethical approval was not necessary under the Polish legal system (Case number: KB nr 946/19).

### Statistical analysis

Data were analyzed using IBM SPSS (version 27.0) and IBM SPSS AMOS (version 26.0). The psychometric properties of the proposed Polish version of the CSAS were evaluated with statistical tests, as listed below.

Construct validity was determined with exploratory factor analysis. Different variants of this analysis are available, and the literature is not consistent on the best options in terms of extraction methods (principal component analysis, principal axis factoring, maximum likelihood) and rotation methods (orthogonal or oblique). However, the aforementioned methods tend to give resembling results [22]. In this study, principal component analysis with direct oblimin was used, as in the majority of other CSAS validation research papers [12, 15–18]. Adequacy of the sample size was examined with the Keiser-Meyer-Olkin (KMO) Measure of Sampling Adequacy and Bartlett’s test of sphericity. The KMO Measure of Sampling Adequacy should be above 0.50, and Bartlett’s test of sphericity should be significant with *p*-value < 0.05 to consider the sample size adequate. The number of factors to retain was determined with Horn’s parallel analysis. Items were included in their respective factors when they loaded at > 0.40 on one factor and at least 0.10 lower on all remaining factors [16]. Items making negative statements about learning communication skills were reversed before the analysis.

The obtained factorial structure was subsequently tested using the confirmatory factor analysis with following fit indices: the minimum discrepancy divided by its degrees of freedom (CMIN/DF), the goodness of fit index (GFI), the adjusted goodness of fit index (AGFI), the normed fit index (NFI), the incremental fit index (IFI), the Tucker-Lewis Index (TLI), the comparative fit index (CFI), the root mean square error of approximation (RMSEA), the Akaike Information Criterion (AIC) and the Bayesian Information Criterion (BIC).

Internal consistency was determined using the Cronbach alpha coefficient with values of alpha > 0.70 considered as acceptable.

Correlation analysis was performed with Spearman’s correlation coefficient between proposed CSAS factors and the total CSAS score.

Due to the lack of other Polish measurement tools related to communication skills learning, assessment of convergent and discriminant validity was not possible.

Test-retest reliability of answers given with an interval of three to 4 weeks was assessed for individual items using weighted kappa coefficients and for subscales with intraclass correlation coefficients (ICC).

## Results

### Demographic characteristic of participants

Out of 440 medical students invited to participate in the study, 400 returned the first copies of questionnaires (90.91% response rate), and 389 (88.41%) of them were completed satisfactorily (contained no missing data in any of CSAS items). Among the respondents who completed the questionnaire satisfactorily, 234 (60.15%) were female, 142 (36.50%) male, and 13 (3.34%) did not disclose their gender. The age of respondents ranged from 18 to 28 (mean = 19.74; median = 19; mode = 19; interquartile range = 19–20).

### Factor structure and internal consistency

The Kaiser-Meyer-Olkin measure of sampling adequacy was 0.873, and Bartlett’s test of sphericity presented a significant *p*-value of < 0.001, both indicating that sample size is adequate, and further analysis could be conducted. The initial factor analysis revealed seven factors with eigenvalues greater than 1, accounting for 56.048% of the variance in data (Table 2).

**Table 2:** Factors with eigenvalues greater than 1

Component	Initial Eigenvalues		
	Total	% of Variance	Cumulative %
1	6.503	25.011	25.011
2	1.740	6.694	31.705
3	1.542	5.931	37.635
4	1.461	5.620	43.255
5	1.240	4.768	48.023
6	1.064	4.093	52.116
7	1.022	3.931	56.048

**Fig. 1:**
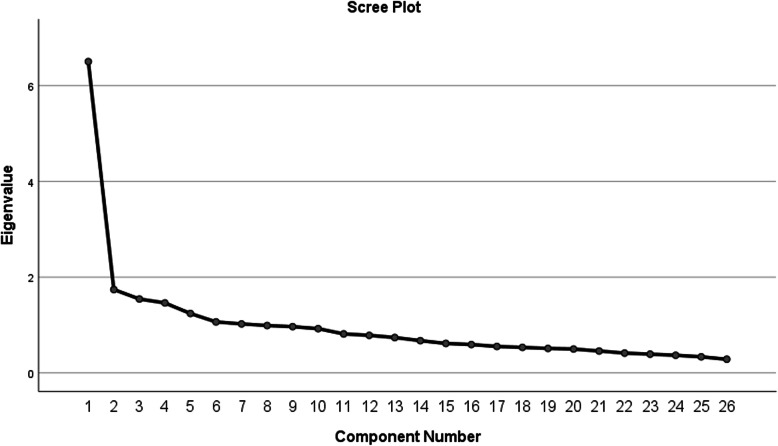
Scree plot with eigenvalues for every component

**Fig. 2:**
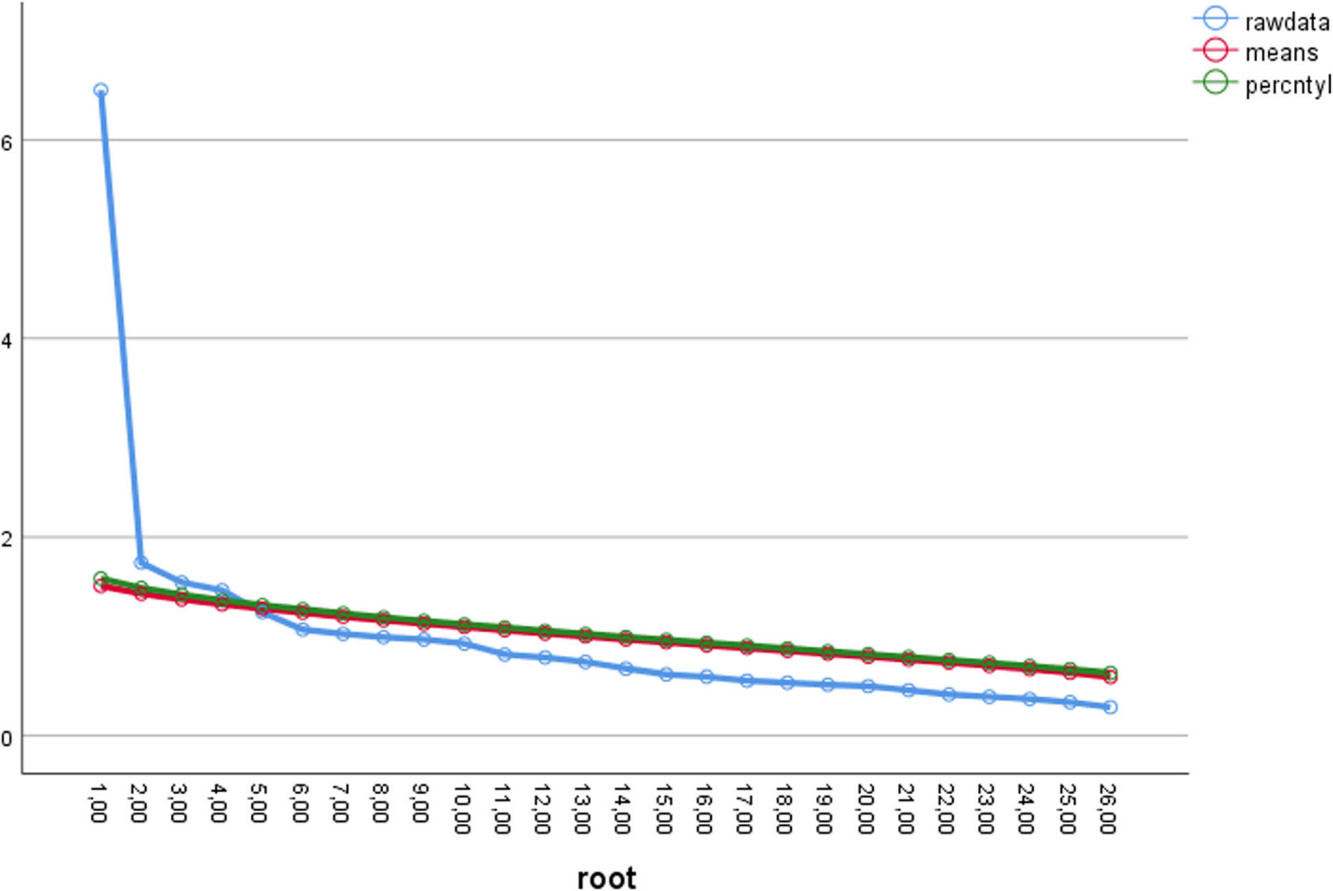
Scree plot with Parallel Analysis results

Analysis of the scree plot (Fig. 1) revealed that the slope leveled off twice, suggesting either two or four factors to retain. Given the subjectivity and criticism of the scree test, Horn’s parallel analysis was performed to specify the number of factors (Fig. 2) [19]. Four factors had eigenvalues greater than mean eigenvalues generated from random data matrices. As a result, they were retained, and the exploratory factor analysis procedure was repeated with the number of factors fixed to four.

Given a strong theoretical basis for the division of the CSAS into two subscales provided by Rees et al. [11], we also attempted to replicate the two-factor solution as proposed by them in another exploratory factor analysis. Detailed results of factor loadings on the pattern matrix after fixing the number of factors to either four or two are presented in Table 3.

**Table 3:** Descriptive Statistics and Pattern Matrixes of both solutions

	Descriptive statistics		Factor loadings on the pattern matrix					
	Mean	SD	**of the four factors**				**of the two factors**	
			1	2	3	4	1	2
Item 5	4.36	0.724	**0.738**	0.059	0.043	0.026	**0.719**	0.002
Item9	4.29	0.746	**0.584**	−0.032	0.298	−0.027	**0.697**	− 0.063
Item10	4.37	0.686	**0.665**	−0.160	0.236	0.194	**0.820**	−0.140
Item14	4.16	0.732	**0.761**	0.148	−0.108	−0.084	**0.624**	0.040
Item16	3.87	0.911	**0.603**	0.085	−0.259	0.093	**0.456**	0.029
Item 4	3.30	1.117	−0.044	**0.554**	−0.104	0.359	−0.079	**0.675**
Item 6^a^	3.64	1.000	0.000	**0.562**	0.255	0.025	0.051	**0.614**
Item 7	3.62	0.954	0.131	**0.670**	0.116	−0.066	0.067	**0.667**
Item12	3.20	0.838	0.158	**0.670**	−0.054	−0.074	0.007	**0.642**
Item18	3.12	1.057	0.140	**0.619**	0.032	0.010	0.062	**0.628**
Item22^a^	2.63	1.098	−0.244	**0.591**	−0.064	0.175	−0.302	**0.678**
Item11^a^	3.49	0.801	0.014	0.235	**0.467**	−0.056	0.195	0.275
Item13^a^	3.52	0.775	0.000	−0.039	**0.572**	−0.129	0.253	−0.016
Item17^a^	3.63	0.972	−0.080	−0.111	**0.566**	0.023	0.225	−0.034
Item25	4.31	0.730	0.211	0.203	**0.442**	0.303	**0.468**	0.335
Item26^a^	4.16	0.803	0.296	0.049	**0.455**	0.157	**0.538**	0.124
Item1	4.45	0.711	0.260	−0.097	0.053	**0.616**	**0.449**	0.077
Item3^a^	3.17	1.100	0.076	−0.002	−0.155	**0.559**	0.143	0.151
Item19^a^	3.80	1.037	−0.033	0.275	−0.017	**0.681**	0.100	**0.499**
Item2^a^	4.58	0.644	0.243	0.127	0.320	0.282	**0.444**	0.233
Item8^a^	2.91	1.053	0.333	0.372	−0.091	−0.224	0.158	0.267
Item15^a^	3.37	0.990	−0.010	0.071	0.345	0.080	0.171	0.139
Item20^a^	3.41	1.060	−0.084	0.002	0.079	0.359	0.054	0.133
Item21	4.04	0.789	0.324	0.404	0.264	0.141	0.416	0.459
Item23	3.73	0.857	0.378	0.018	0.036	0.332	**0.460**	0.092
Item24^a^	3.66	0.916	0.056	0.498	0.419	−0.036	0.178	**0.542**

^a^ items making negative statements about learning communication skills that were reversed before the analysis

Extraction Method: Principal Component Analysis

Rotation Method: Oblimin with Kaiser Normalization

As can be observed in Table 3, in the case of the four-factor solution, five items loaded on Factor 1 and all of them were making positive statements about learning communication skills (items 5, 9, 10, 14, 16). Since they all describe outcomes that students expect to gain from learning communication skills, we named this factor “Perceived outcomes.”

Six items loaded on Factor 2. Four of them were making positive statements about learning communication skills (items 4, 7, 12, 18), and two of them were making negative statements (items 6 and 22). Since most items involved positive attitudes towards communication skills learning and previously reversed items making negative statements, this factor was named “Positive Attitudes Towards Communication Learning (CL).”

Five items loaded on Factor 3. One of them was making a positive statement about learning communication skills (item 25), and four were making negative statements (items 11, 13, 17, and 26). Since the majority of items denoted negative attitudes and prejudices towards communication skills learning, this factor was named “Negative Attitudes Towards Communication Learning (CL).”

Three items loaded on Factor 4. One was making a positive statement about learning communication skills (item 1), and two were making negative statements (items 3 and 19). Since they describe things that motivate (or demotivate) students to pursue communication skills learning, this factor was named “Motivation.”

Items included in each factor and their wording are presented in Table 4.

**Table 4:** Four factors with corresponding items

Factor 1 - PERCEIVED OUTCOMES (α = 0.758) 5. Learning communication skills has helped or will help me respect patients 9. Learning communication skills has helped or will help facilitate my team-working skills 10. Learning communication skills has improved (or will improve) my ability to communicate with patients 14. Learning communication skills has helped or will help me respect my colleagues 16. Learning communication skills has helped or will help me recognise patients’ rights regarding confidentiality and informed consent	
Factor 2 - POSITIVE ATTITUDES TOWARDS CL (α = 0.743) 4. Developing my communication skills is just as important as developing my knowledge of medicine 6.^a^ I haven’t got time to learn communication skills 7. Learning communication skills is interesting 12. Learning communication skills is fun 18. When applying for medicine, I thought it was a really good idea to learn communication skills 22.^a^ My ability to pass exams will get me through medical school rather than my ability to communicate	
Factor 3 - NEGATIVE ATTITUDES TOWARDS CL (α = 0.557) 11.^a^ Communication skills teaching states the obvious and then complicates it 13.^a^ Learning communication skills is too easy 17.^a^ Communication skills teaching would have a better image if it sounded more like a science subject 25. Learning communication skills is important because my ability to communicate is a lifelong skill 26.^a^ Communication skills learning should be left to psychology students, not medical students	
Factor 4 - MOTIVATION (α = 0.535) 1. In order to be a good doctor I must have good communication skills 3.^a^ Nobody is going to fail their medical degree for having poor communication skills 19.^a^ I don’t need good communication skills to be a doctor	

^a^ items making negative statements about learning communication skills that should be reversed before statistical analysis

Items are presented in English based on the original CSAS: Rees C, Sheard C, Davies S. The development of a scale to measure medical students' attitudes towards communication skills learning: the Communication Skills Attitude Scale (CSAS). Medical Education. 2002; 36(2):141-7. John Wiley and Sons (© Blackwell Science Ltd)

In the case of the two-factor solution, obtained factor loadings did not correspond satisfactorily with the proposed PAS and NAS subscales structure, and their naming was problematic. Ten items loaded on Factor 1. Eight of them were making positive statements about learning communication skills (items 1, 5, 9, 10, 14, 16, 23, 25), and two were previously reversed items with negative statements (2 and 26). Eight items loaded on Factor 2. Four of them were making positive statements about learning communication skills (items 4, 7, 12, 18), and four were previously reversed items with negative statements (6, 19, 22, and 24). In so far as the first subscale can be roughly perceived as corresponding with the original PAS counterpart, the second subscale containing equal numbers of positive and negative statements cannot really be considered as the counterpart of NAS.

**Fig. 3:**
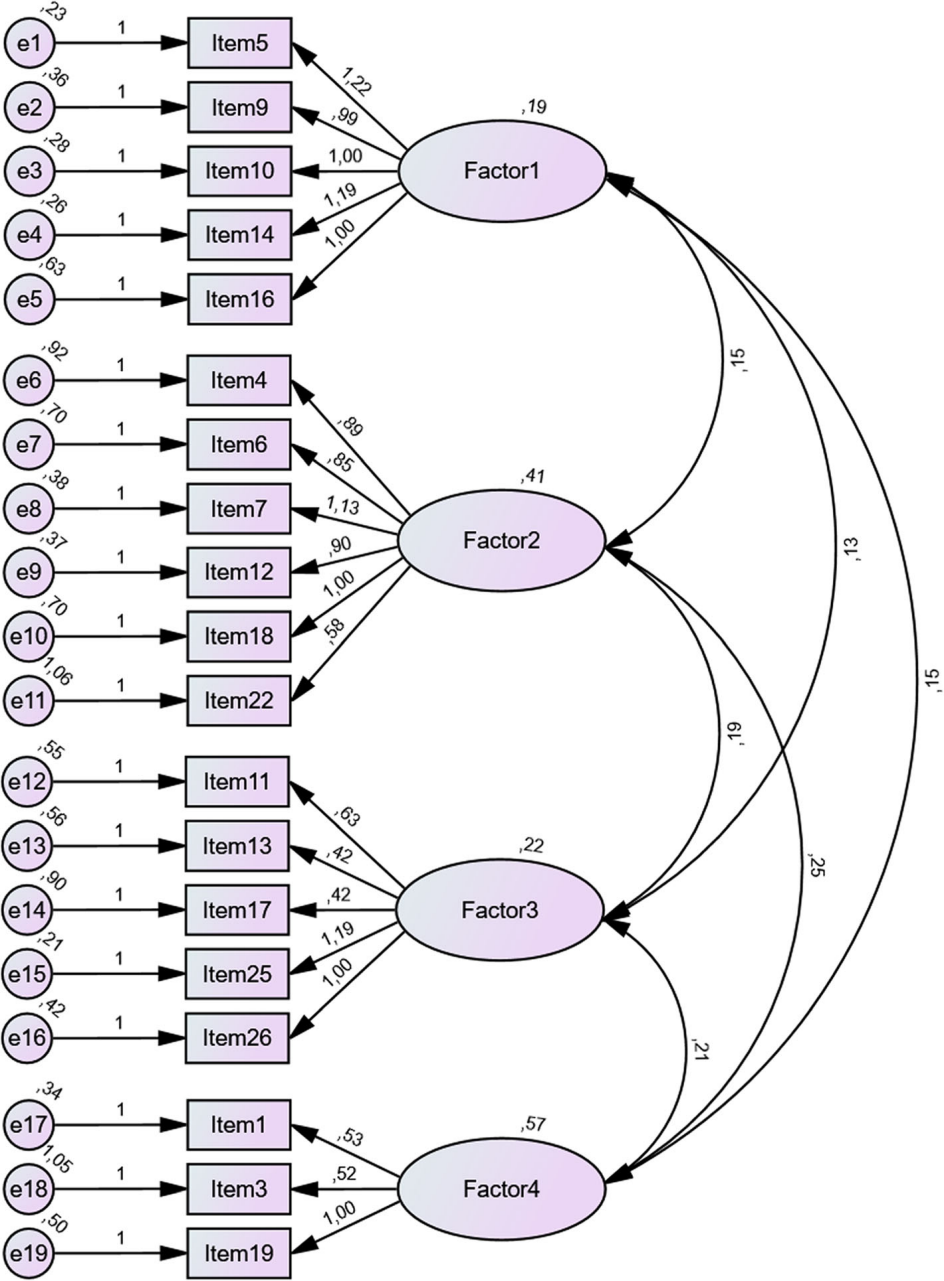
Best-fitting (four-factor) model - results from CFA

Confirmatory factor analysis (CFA) was conducted to compare the four-factor solution obtained during exploratory factor analysis with the aforementioned two-factor solution and the original model with division into PAS and NAS as proposed by Rees et al. [11]. Results obtained indicated poorer fit of both two-factor models, confirming that the four-factor model would be more appropriate (Fig. 3). Detailed results of the confirmatory analysis of all three variants of the CSAS are presented in Table 5.

**Table 5:** Comparison of fit indexes of three CSAS models

	Better fit model	Four-factor CSAS	Two-factor CSAS	Original CSAS (with data from this study)
CMIN/DF	lower values	2.738	4.041	3.329
GFI	closer to 1.000	0.898	0.860	0.818
AGFI	closer to 1.000	0.867	0.821	0.786
NFI	closer to 1.000	0.789	0.747	0.650
IFI	closer to 1.000	0.855	0.797	0.727
TLI	closer to 1.000	0.828	0.766	0.699
CFI	closer to 1.000	0.853	0.795	0.724
RMSEA	lower values	0.067	0.089	0.077
AIC	lower values	487.764	615.543	1097.898
BIC	lower values	662.161	762.195	1307.967

*CMIN/DF* The minimum discrepancy divided by its degrees of freedom

*GFI* The goodness of fit index

*AGFI* The adjusted goodness of fit index

*NFI* The normed fit index

*IFI* The incremental fit index

*TLI* The Tucker-Lewis Index

*CFI* The comparative fit index

*RMSEA* The root mean square error of approximation

*AIC* The Akaike Information Criterion

*BIC* The Bayesian Information Criterion

### Internal consistency

The Cronbach’s alpha values of individual subscales were as follows: 1 - Perceived outcomes (α = 0.758); 2 - Positive Attitudes Towards CL (α = 0.743); 3 - Negative Attitudes Towards CL (α = 0.557) and 4 - Motivation (α = 0.535) (Table 4). Since the Cronbach’s alpha values of the subscales 3 and 4 are lower than the acceptable value, we recommend caution when using them alone. However, in our opinion, they are still worthy of inclusion given the number of items in them and the observation that Cronbach’s alpha tends to depend on the number of items in a subscale [23]. The Cronbach’s alpha value of the total CSAS scale (including all 26 items) was equal to 0.853.

### Correlation analysis

All proposed CSAS subscales correlate significantly with the total CSAS score (Spearman’s correlation coefficient between 0.564 and 0.812), and to a lesser extent, with each other (Spearman’s correlation coefficient between 0.201 and 0.416) (Table 6). It can also be noted that correlations between subscales “Negative Attitudes Towards CL” and “Motivation” and other subscales are weaker.

**Table 6:** Correlations between proposed factors and total CSAS score

	Total CSAS	Factor 1	Factor 2	Factor 3	Factor 4
**Total CSAS**	1.000	0.700^a^	0.812^a^	0.603^a^	0.564^a^
**Factor 1 - Perceived outcomes**	0.700^a^	1.000	0.416^a^	0.328^a^	0.311^a^
**Factor 2 - Positive Attitudes Towards CL**	0.812^a^	0.416^a^	1.000	0.356^a^	0.372^a^
**Factor 3 - Negative Attitudes Towards CL**	0.603^a^	0.328^a^	0.356^a^	1.000	0.201^a^
**Factor 4 - Motivation**	0.564^a^	0.311^a^	0.372^a^	0.201^a^	1.000

^a^ Correlation is significant at the 0.01 level (2-tailed)

### Test-retest reliability

Seventy-one students (45 females and 26 males) completed the second questionnaire and no items were missing. Unfortunately, we were not able to reach the desired “excellent” threshold of at least 100 respondents for the second questionnaire, but the sample size between 50 and 99 is still considered as “good” [24]. Levels of agreement indicated by weighted kappa coefficients were substantial or moderate in case of most items (22; 84.62%) which denotes satisfactory test-retest reliability (Table 7). The test-retest analysis measured by intraclass correlation coefficients for individual subscales were as follows: Factor 1 - Perceived outcomes - ICC = 0.876 (95% CI 0.802–0.923, *p* < 0.001); Factor 2 - Positive Attitudes Towards CL - ICC = 0.868 (95% CI 0.788–0.918, *p* < 0.001); Factor 3 - Negative Attitudes Towards CL - ICC = 0.858 (95% CI 0.772–0.911, *p* < 0.001); Factor 4 - Motivation - ICC = 0.801 (95% CI 0.681–0.876, *p* < 0.001).

**Table 7:** Test-retest reliability results of individual items

Weighted Kappa	Levels of agreement^a^	THIS STUDY		Original CSAS^a^	
		Frequency	Item number	Frequency	Item number
0.61–0.80	Substantial	6 (23.08%)	4, 11, 14, 19, 22, 26	1 (3.85%)	18
0.41–0.60	Moderate	16 (61.54%)	1, 5, 6, 7, 8, 9, 12, 15, 16, 17, 18, 20, 21, 23, 24, 25	17 (65.38%)	2, 3, 4, 5, 7, 8, 9, 10, 11, 12, 14, 17, 19, 20, 21, 22, 24
0.21–0.40	Fair	3 (11.54%)	2, 3, 10	7 (26.92%)	6, 13, 15, 16, 23, 25, 26
0.00–0.20	Slight	1 (3.85%)	13	1 (3.85%)	1

^a^ as reported by Rees et al. [11]

## Discussion

The presented study offers some more evidence for the validity and reliability of the Communication Skills Attitude Scale (CSAS). Although the two-factor model of the CSAS was supported by many studies [12–15], results from this analysis demonstrate that in the examined population, the four-factor solution may be more suitable. As mentioned above, other researchers also previously described some poly-factorial solutions of the CSAS, including three-factorial [16], four-factorial [17] and five-factorial [18]. However, their decisions were mainly based on less recommended Kaiser criterion and analysis of the scree plot. In contrast, we decided to supplement the scree plot test with the highly recommended Horns’ parallel analysis (PA) to assess the number of factors that should be retained.

Moreover, the four factors received in this study seem to reflect Polish conditions appropriately. On the one hand, two factors evaluate students’ positive and negative attitudes towards communication training, representing their feelings on the learning process itself and how it is organized. On the other hand, Polish students seem to differentiate between their attitudes towards communication learning and two other factors, namely its perceived outcomes and their motivation towards it. This division may reflect the differences in the Polish medical education system described above. The lower emphasis on communication training than in some other countries might, due to the demanding nature of the studies and lack of time, reduce students’ motivation compared to other courses, where they have to pass an exam, for instance. Simultaneously, it does not necessarily mean that they perceive communicating with patients as unimportant or do not see positive outcomes of its training. Finally, they may dislike particular training methods or the way classes are conducted, for example, expecting more experiential training methods instead of lectures and seminars. All in all, given the shape of the Polish medical curriculum, their attitudes in respect to the aforementioned factors may vary. Some of the factors described in this study also seem to be more universal, appearing in different variants in other poly-factorial solutions (Table 1). For instance, the “Learning” factor appears in the Norwegian [16] and Iranian [17] CSAS, both representing students’ attitudes towards communication learning itself. Similarly, our “Perceived outcomes” seem comparable with the “Respecting” factor in Norwegian CSAS [16] and “Facilitating interpersonal skills” in the Korean CSAS [18]. Meanwhile, counterparts of some other factors were not found in our study, for example those pertaining to students’ negative attitudes towards the assessment of communication skills from the Korean solution [18] or excuses for lack of participation in communication training from the Iranian CSAS [17]. It may serve as yet another example of the specificity of Polish conditions. Given that the medical studies are regulated by the Ministry, realization of all imposed learning outcomes is obligatory for graduation. Consequently, an individual student cannot simply decide not to participate in a course that is marked as compulsory in the curriculum. Moreover, although students participating in the study will be the first year whose communication skills will be formally assessed during their studies, they might only be getting used to this change and as a result it was not reflected in a separate factor.

As a result, a four-factorial structure of the CSAS was imposed (Table 4). Seven items failed to successfully load on any factor (items 2, 8, 15, 20, 21, 23, 24) and were subsequently eliminated. As it can be observed in Table 1**,** other studies also report different numbers of items eliminated due to unsatisfactory or ambiguous factor loadings. Furthermore, it should be highlighted that in this study, as well as many others, the inclusion of individual items into their factors was made on some a priori grounds (usually high loadings on one factor and low or no ambiguous loadings on others). On the other hand, Rees et al. [11] decided to include all items based on theoretical assumptions. Moreover, at least some discrepancies between item loadings observed in different CSAS validation studies can also be attributed to the cultural and language differences as described above.

Table 1 shows that among various constructs, our results are most comparable with those of Anvik et al. [16] even though their model involves three factors and our four factors. Their biggest factor, “Learning,” might be roughly regarded as a composite of both our factors associated with attitudes towards communication learning (Factors 2 and 3). Similarly, their factors “Importance” and “Respecting” are comparable with our “Motivation” and “Perceived outcomes,” respectively. This supports their suggestion that the CSAS may be able to differentiate between two components of attitudes - affective and cognitive. The affective component in their study was represented by the factor “Learning,” while in our study, as described before, it is divided into two factors (“Positive Attitudes Towards CL” and “Negative Attitudes Towards CL”). In the case of the cognitive component, in their study, it was represented by the factor “Importance,” whereas in the present study, it is named “Motivation.” As Anvik et al. [16] rightly noted, *“this is important because affective attitudes are easily influenced by experience while cognitive attitudes are more basic and stable. Negative affective attitudes towards learning communication skills may signal that students perceive the way skills are taught negatively, but does not necessarily mean negative attitudes towards the benefit of using such skills when seeing patients.”* In fact, results of this study demonstrate that Factors 3 and 4 seem to correlate weaker with each other and remaining factors. Furthermore, in this aspect the aforementioned Ajzen’s Theory of Planned Behavior [10] should be consulted. It assumes that one’s intentions directly influence their behavior and are determined by attitudes, subjective norms and perceived behavioral control. Attitudes denote an extent to which given behavior is perceived as favorable or unfavorable as well as beliefs in its expected outcomes, and this variable seems to be covered satisfactorily by the CSAS. However, other two components remain practically unexplored. Briefly, subjective norms reflect personally perceived social pressure to perform or cease to perform the behavior and perceived behavioral control indicates one’s ease or difficulty to perform given behavior, including past experiences and predicted obstacles. It may be therefore beneficial to additionally develop a measurement tool covering all three aforementioned variables.

Given the theoretical basis provided by Rees et al. [11] and research protocols of other authors, we also decided to recreate the two-factorial structure of the CSAS. Noteworthy, to the best knowledge of authors, this is the first study to report a direct comparison of a poly-factorial CSAS model with models based on theoretical assumptions (two factors). Results of the confirmatory factor analysis (CFA) indicated a better fit of the four-factor model in comparison with both two-factor models, including the original subscales presented by Rees et al. [11]. This study is among the few papers on the CSAS validation to perform CFA [14, 17], so chances for comparison of results are limited. It should be also mentioned that it was conducted with a small sample size, smaller than in aforementioned studies, which may have affected the results and we recommend caution in the interpretation. Busch et al. [14] received an assumable model after improving the model fit with modification indexes, something that we decided to avoid and present our data unchanged. Another confirmation of the proposed CSAS in CFA comes from a recent study examining the four-factor model [17].

Results of the test-retest reliability analysis indicate good stability of the Polish CSAS among medical students. Weighted kappa coefficients indicating substantial or moderate levels of agreement were detected in most items (22; 84.62%), suggesting satisfactory and comparable results with those of Rees et al. [11]. The test-retest analysis of individual subscales also yielded acceptable results with intraclass correlation coefficients (ICC) ranging from 0.801 (Factor 4 - Motivation) to 0.876 (Factor 1 - Perceived outcomes). Due to differences in factorial structure, it is difficult to compare them. In the case of Rees et al. [11], the ICC values were equal to 0.646 (PAS) and 0.771 (NAS) and in one known test-retest reliability analysis for a four-factorial model of the CSAS ICC values ranged from 0.67 to 0.87 [17], which seems quite comparable with our results. However, in their case, only 20 students filled the retest questionnaire. For comparison, the original CSAS validation retest procedure was performed on 39 students [11], and in our case, it involved 71 students. It should be noted that the test-retest interval in this study was longer than the usual two-week interval in the majority of papers published so far. This is important given that if this interval is too short, students may simply remember their previous answers, and if it is too long, students’ attitudes may change in the meantime [25].

### Limitations

We acknowledge limitations of the study. Firstly, although presented results indicate good overall internal consistency of the CSAS scale (α = 0.853) and two subscales (α = 0.758 and 0.743), the Cronbach’s alpha values of the remaining two subscales (α = 0.557 and 0.535) are lower than the acceptable value. Still, as presented in Table 1**,** our results seem to be comparable with those of other authors, especially in the CSAS models with more than two factors. Moreover, as we mentioned above, it was recognized that Cronbach’s alpha value is dependent on the number of items in a subscale [23]. Generally, scales with fewer items give lower Cronbach’s alpha coefficients, which may explain the results of this paper and others. Furthermore, across many studies, the NAS subscale and its counterparts present lower internal consistency than the PAS subscale, which is also replicated in our results. A second limitation of the study is the small sample of students for the test-retest reliability. Although the sample of 71 students can still be considered as “good” [24], a considerable drop is noticeable in comparison with the sample of students who completed the first questionnaire. Students were reminded twice about completing the second questionnaire. Given the anonymous and voluntary character of the study, we did not inquire in detail why the remaining students did not return the second copy. However, some students reported losing it and the rest probably did not find the time or did not want to. Thirdly, the study was conducted on students of only one medical school and involved only 1 year. The reason for that was to ensure that no communication-specific content was conveyed in between the period of test-retest procedure that might have influenced students’ attitudes. As a result, we see the need for further studies involving different medical schools and study years. Finally, the number of female respondents was higher than males. However, this is consistent with the general population of the first year medical students of our University equal to 274 (62.27%) females and 166 (37.73%) males.

## Conclusions

To conclude, this study provided some more evidence for the validity and reliability of the CSAS and demonstrated a validated version of the CSAS for Polish medical students. Despite the popularity and theoretical basis, the division of the CSAS into two factors may not always be the most appropriate solution. As previously suspected, attitudes of medical students towards communication skills may transgress simple categorization into positive and negative. Different components of their attitudes should be considered. There may be a difference between students’ attitudes towards learning communication skills and their opinions on its potential outcomes, difficulties, or perceived expectations of society. The CSAS seems appropriate to differentiate also between affective and cognitive components of students’ attitudes. Given that according to Ajzen’s Theory of Planned Behavior, an intention to perform a behavior is conditioned not only on attitudes but also on subjective norms and perceived behavioral control, to assess the situation fully, there may be a need to design a measurement tool covering all aforementioned variables.

## Supplementary Information

**Additional file 1:.** Polish and the original English version of the CSAS scale. Translated and adapted version of the CSAS questionnaire in Polish. The Polish version of the CSAS, which was developed for the purpose of this study, was also supplemented with the original English version as presented by Rees et al. [11].

## Data Availability

The datasets used and/or analyzed during the current study are available from the corresponding author on reasonable request.

## Abbreviations

CSAS: Communication skills attitude scale
PAS: Positive attitudes scale
NAS: Negative attitudes scale
PA: Horns’ parallel analysis
CFA: Confirmatory factor analysis
CMIN/DF: The minimum discrepancy divided by its degrees of freedom
GFI: The goodness of fit index
AGFI: The adjusted goodness of fit index
NFI: The normed fit index
IFI: The incremental fit index
TLI: The tucker-lewis index
CFI: The comparative fit index
RMSEA: The root mean square error of approximation
AIC: The akaike information criterion
BIC: The bayesian information criterion
ICC: Intraclass correlation coefficient
CL: Communication learning
