# Supplementary material for: Affective and cognitive components of students’ attitudes towards communication learning - validation of the Communication Skills Attitude Scale in a cohort of polish medical students
Source: BMC Med Educ. 2021 Apr 1;21:190. doi: 10.1186/s12909-021-02626-7 (PMC8017827; doi:10.1186/s12909-021-02626-7)
Supplement: Supplementary file 1 — Additional file 1:. Polish and the original English version of the CSAS scale. Translated and adapted version of the CSAS questionnaire in Polish. The Polish version of the CSAS, which was developed for the purpose of this study, was also supplemented with the original English version as presented by Rees et al. [11]. [file 12909_2021_2626_MOESM1_ESM.pdf]

Additional file 1. Polish and the original English version of the CSAS scale.

**Proszę o zapoznanie się z poniższymi stwierdzeniami dotyczącymi uczenia się umiejętności komunikacyjnych oraz wskazanie odpowiedzi najbardziej pasującej do Państwa opinii zgodnie z następującą skalą:**<br>**1 = zdecydowanie się nie zgadzam**<br>**2 = nie zgadzam się**<br>**3 = nie mam zdania**<br>**4 = zgadzam się**<br>**5 = zdecydowanie się zgadzam**<br>[Please read the following statements about communication skills learning. Indicate whether you agree or disagree with all of the statements by circling the most appropriate response. Remember,<br>1 = strongly disagree<br>2 = disagree<br>3 = neutral<br>4 = agree<br>5 = strongly agree]*						
1	**Aby być dobrym lekarzem, muszę mieć dobre umiejętności komunikacyjne**<br>[In order to be a good doctor I must have good communication skills]	1	2	3	4	5
2	**Nie widzę sensu w uczeniu się umiejętności komunikacyjnych**<br>[I can't see the point in learning communication skills]	1	2	3	4	5
3	**Słabe umiejętności komunikacyjne nie stanowią przeszkody w ukończeniu studiów na kierunku lekarskim**<br>[Nobody is going to fail their medical degree for having poor communication skills]	1	2	3	4	5
4	**Rozwijanie umiejętności komunikacyjnych jest dla mnie tak samo ważne jak rozwijanie wiedzy medycznej**<br>[Developing my communication skills is just as important as developing my knowledge of medicine]	1	2	3	4	5
5	**Uczenie się umiejętności komunikacyjnych pomogło lub pomoże mi odnosić się z szacunkiem do pacjentów**<br>[Learning communication skills has helped or will help me respect patients]	1	2	3	4	5
6	**Nie mam czasu, żeby uczyć się umiejętności komunikacyjnych**<br>[I haven't got time to learn communication skills]	1	2	3	4	5
7	**Uczenie się umiejętności komunikacyjnych jest ciekawe**<br>[Learning communication skills is interesting]	1	2	3	4	5
8	**Nie powinno się mnie zmuszać do przychodzenia na zajęcia dotyczące umiejętności komunikacyjnych**<br>[I can't be bothered to turn up to sessions on communication skills]	1	2	3	4	5
9	**Uczenie się umiejętności komunikacyjnych polepszyło lub polepszy moje umiejętności pracy w zespole**<br>[Learning communication skills has helped or will help facilitate my team-working skills]	1	2	3	4	5
10	**Uczenie się umiejętności komunikacyjnych poprawiło (lub poprawi) moją umiejętność rozmowy z pacjentami**<br>[Learning communication skills has improved my ability to communicate with patients]	1	2	3	4	5

11	**Zajęcia z umiejętności komunikacyjnych stwierdzają rzeczy oczywiste, a następnie je komplikują**<br>[Communication skills teaching states the obvious and then complicates it]	1	2	3	4	5
12	**Uczenie się umiejętności komunikacyjnych to przyjemność**<br>[Learning communication skills is fun]	1	2	3	4	5
13	**Uczenie się umiejętności komunikacyjnych jest za łatwe**<br>[Learning communication skills is too easy]	1	2	3	4	5
14	**Uczenie się umiejętności komunikacyjnych pomogło lub pomoże mi odnosić się z szacunkiem do współpracowników**<br>[Learning communication skills has helped or will help me respect my colleagues]	1	2	3	4	5
15	**Ciężko mi zaufać informacjom odnośnie umiejętności komunika-cyjnych udzielnych mi przez osoby niezwiązane z pracą kliniczną**<br>[I find it difficult to trust information about communication skills given to me by non-clinical lecturers]	1	2	3	4	5
16	**Uczenie się umiejętności komunikacyjnych pomogło lub pomoże mi honorować prawa pacjentów dotyczące poufności i świadomej zgody**<br>[Learning communication skills has helped or will help me recognise patients' rights regarding confidentiality and informed consent]	1	2	3	4	5
17	**Zajęcia uczące komunikacji miałyby lepszy wizerunek, gdyby ich nazwa brzmiała w sposób bardziej naukowy**<br>[Communication skills teaching would have a better image if it sounded more like a science subject]	1	2	3	4	5
18	**Starając się o przyjęcie na studia medyczne uznałem, że dobrym pomysłem będzie nauka umiejętności komunikacyjnych**<br>[When applying for medicine, I thought it was a really good idea to learn communication skills]	1	2	3	4	5
19	**Nie potrzebuję dobrych umiejętności komunikacyjnych, aby zostać lekarzem**<br>[I don't need good communication skills to be a doctor]	1	2	3	4	5
20	**Trudno mi przyznać się, że mam pewne problemy z moimi umiejętnościami komunikacyjnymi**<br>[I find it hard to admit to having some problems with my communication skills]	1	2	3	4	5
21	**Uważam, że uczenie się umiejętności komunikacyjnych w trakcie studiów medycznych jest bardzo przydatne**<br>[I think it's really useful learning communication skills on the medical degree]	1	2	3	4	5
22	**Umiejętność zdawania egzaminów jest bardziej przydatna do ukończenia studiów medycznych niż umiejętności komunikacyjne**<br>[My ability to pass exams will get me through medical school rather than my ability to communicate]	1	2	3	4	5
23	**Uczenie się umiejętności komunikacyjnych ma zastosowanie w nauce medycyny**<br>[Learning communication skills is applicable to learning medicine]	1	2	3	4	5
24	**Ciężko mi traktować na poważnie uczenie się umiejętności komunikacyjnych**<br>[I find it difficult to take communication skills learning seriously]	1	2	3	4	5

25	**Uczenie się umiejętności komunikacyjnych jest ważne, ponieważ są to umiejętności na całe życie** [Learning communication skills is important because my ability to communicate is a lifelong skill]	1	2	3	4	5
26	**Uczenie się komunikacji powinno być zostawione studentom psychologii, a nie medycyny** [Communication skills learning should be left to psychology students, not medical students]	1	2	3	4	5
Opracowano w oparciu o [*Developed on the basis of*]: Rees C, Sheard C, Davies S. The development of a scale to measure medical students' attitudes towards communication skills learning: the Communication Skills Attitude Scale (CSAS). Medical Education. 2002; 36(2):141-7. John Wiley and Sons (© Blackwell Science Ltd)						

*Polish version of the CSAS is presented in bold font. The original English version of the CSAS is given in square brackets.
